# Supplementary material for: How does moving public engagement with research online change audience diversity? Comparing inclusion indicators for 2019 & 2020 European Researchers’ night events
Source: PLoS One. 2022 Mar 11;17(3):e0262834. doi: 10.1371/journal.pone.0262834 (PMC8916624; doi:10.1371/journal.pone.0262834)
Supplement: Attachment — Submitted filename: Reviewers comments.docx [file pone.0262834.s001.docx]

**How Does Moving Public Engagement with Research Online Affect Audience Diversity? Comparing Inclusion Indicators for 2019 & 2020 European Researchers’ Night Events**

**General comments**

The manuscript is technically sound, well-written, and all data and statistics are clearly presented. It demonstrates an important finding, that shifting public science events online can polarise the audience by strengthening attendance from higher educated groups whilst also attracting those with no formal qualification. I felt that the manuscript was rather too simplistic in this analysis however, as there was no discussion about other interesting demographics and how these may be affected by a move to an online delivery. It was also not explained why Malta and Ireland were chosen for this study, and no recommendations were given for improving online events to attract a wider demographic. I would be interested to see a revised version that presents evidence for future event organisers (and other science communication practitioners) to use to ensure greater representation of diverse society at these events.

Detailed comments are below:

**Abstract:** suggest numbering your first and second hypotheses to be clear that these are discreet, and improve the flow for the reader.

**European Researchers’ Night: Background and Changing Context:**

I suggest this would be a good place to explain why Malta and Ireland are the focus of this study. This is not discussed anywhere in the manuscript. Information is provided for each country, but not an explanation of why these countries were selected, which seems crucial.

**ERN in Malta:**

The sentence ‘In Malta in 2019, European Researchers’ Night was reported as the largest national science and arts festival, attracting around 30,000 people’ is not clear whether this means the largest national science event in Malta, or the largest event across all European cities with ERNs. Is this why Malta was selected for the study? Please clarify.

I note an interesting discrepancy that Malta attracted considerably fewer viewers in 2020 compared to the 2019 face-to-face event, whilst across all European countries there were much higher rates of participation in 2020. What is the reason for this perhaps? Is it important? It’s not discussed.

**ERN in Ireland:**

It appears from this section that the programme of events between Ireland and Malta is very different and this needs some discussion. Malta appeared to have a more interactive, entertainment-style series of events compared particularly to Cork in 2019. There seemed to be more interactive/storytelling style events in Dublin in 2019, but both Irish cities were perhaps more creative in 2020 in their online offerings. This seems to be very interesting and likely to have significant consequences, especially if these were advertised differently to attract a different demographic compared to the more formal science debates, information sessions, etc.

No information on participant numbers for Ireland in 2019 and 2020 – how do these compare with Malta, and with the general numbers across Europe?

**Procedure:**

Roughly how long did the surveys take for participants to complete in 2019 and 2020, and were the questions similar for Malta and Ireland? What might the impact of only having these in English language be?

**Sample:**

Table 1 – terms ‘enrolled’ and ‘achieved’ are not clear and need to be explained in the text. Does enrolled mean they signed up for the survey but only the ‘achieved’ participants completed it? If so, there is a huge drop-off rate in 2020 – what implications does this have?

The sentence ‘While a much larger sample frame is indicated in 2020, the survey was carried out differently in each year of data collection’ doesn’t seem to make sense and the following paragraph doesn’t explain what the likely impact of this might be.

The justification for Case 1 and 2 being discreet cases is explained as the organisers changing between 2019 and 2020, but for the other cases was the event programme sufficiently similar even with the same organisers? Or alternatively, was the programme in case 1 and case 2 substantially different between the different organisers? There seem to be a lot of different variables that could impact the comparison between 2019 and 2020, and I see that the organisers may be an important one but not necessarily greater than other programme changes. Can you explain this choice further, with examples of how this was important?

Is the terminology ‘cases’ necessary, as it’s quite confusing? It’s a layer of jargon to interpret. Can you not just state Cork, Dublin and Malta? This terminology is not used beyond this paragraph – was it important for the statistics? It’s not used in the results, so I’m not sure what this refers to.

**Figure 1**

I find this figure very difficult to interpret and am not convinced it adds anything here. The data discussed prior to this reveals interesting differences across each education level, which are not linear, so splitting them into above and below undergraduate doesn’t seem to follow logically.

**Table 7**

This repeats the numbers stated in the text so doesn’t add any new information. Differences in ‘some needs but not all’ are echoed in the ‘all needs or more’ group between years since 100% of respondents fall into these two categories.

**Discussion**

The discussion is rather simplistic and could make some recommendations for how online events may be tailored better to engage a wider demographic? What has been learned about how this worked in Malta compared to Ireland? The reasons for comparing the two countries is not clear, and some direction to inform future events in these countries would be beneficial here. The authors could attempt to explain why higher numbers of people with no formal qualification were attracted to the online events in 2020 compared to 2019. Additionally, is there any evidence of engagement, interest, learning from these events between the two years in addition to simply attendance?

The term ‘profiles of audiences’ is used throughout the manuscript, but is very basic – just general qualification level and a simplistic statement about household economic status. It seems the authors could have also looked into other interesting demographics, such as age and gender.

Line 3 of the discussion ‘several cities’ is misleading as it was just 3 in total.

‘Cross-year comparison of the same public engagement events’ is misleading as the virtual events were very different to the face-to-face versions
