# Supplementary material for: How does moving public engagement with research online change audience diversity? Comparing inclusion indicators for 2019 & 2020 European Researchers’ night events
Source: PLoS One. 2022 Mar 11;17(3):e0262834. doi: 10.1371/journal.pone.0262834 (PMC8916624; doi:10.1371/journal.pone.0262834)
Supplement: Attachment — Submitted filename: Response to Reviewers.docx [file pone.0262834.s002.docx]

Dear PLOSONE editorial staff,

Please find below our responses to the reviewers' comments.

| Reviewer #1 | | | |
| --- | --- | --- | --- |
| 1 | Summary | This paper adopted a case, the 2019 & 2020 European Researchers’ Night Events, to investigate the digital divide of online and offline participants, however, relying on survey methods. The limitations are obvious, conclusion and implications **can only be understood in a narrow context**, such as the European Researchers’ Night Events. Hence, from my perspective, a qualitative method seems more appropriate for this type of research. | We have already been careful not to make grand conclusions extending beyond European Researchers Night from this study. |
| 2 | Research questions | The research questions including two hypotheses: audience participation in online events seem to have higher educational attainment and subjective economic well-being when compared to those engaged in offline activities, seems can not provide this field with enough contribution and implications. It is more like a course paper than a journal paper, and I recommend you rethink the RQs and Hypos based on what theoretical and practical implications you can provide to this field. | Reviewer opinion but nothing substantive to address. Have expanded on the implications for the field. |
| 3 | Method | The sample method for this second hand survey data, which seems to have adopted a convenience sampling method, can not guarantee representativeness, which in turn undermines the conclusion and generality of your study. | The paper already explicitly notes the sampling method, adopted for practical reasons during this live, real world event, in its current discussion of limitations. As these events do not take place in classroom or laboratory settings, strict sampling controls were not feasible. As we already stated in the methods, “The research approach balanced the practical compromises needed for real-world naturalistic exploratory research and sampling best practices, such as having equal probability of selection as well as random allocation to treatment and control groups. This is often difficult in audience research settings, where public audiences have free choice about where they spend their leisure time [23, 24].” |
| 4 | Statistics | In the data analysis part, more advanced statistical methods could be applied, instead of too descriptive evidence, to show direct and robust evidence for the reader’s understanding. | This is an external critique. More advanced statistical analysis could always be applied to any study. We disagree that employing more advanced inferential statistical tests would aid reader's understanding given this particular dataset. |

| Reviewer #2 | | | | |
| --- | --- | --- | --- | --- |
| 1 | Summary | A descriptive review of the change in audience attending the ERN, an annual European science festival, as it transforms from physical to digital delivery in a pre and post-COVID-19 climate. The study compares two social inclusion metrics within nations Malta and Ireland, namely levels of education attained and self-reported economic well-being. The manuscript clearly delineates between the evening expo events and how online content has compensated with mixed media interactions available in the digital space. The author articulates their hypotheses, methods, and results understandably. | No changes needed. |  |
| 2 |  | Hypotheses of increased educational attainment levels and greater self-reported economic well-being were both upheld and not. In the context of diversity, questions arise whether COVID-19 potentiated skews in education and or economic well-being could be explored further. The manuscript could be strengthened with other measures of diversity and additional examination into reasons for hypotheses not being upheld; if the data is available to support more investigation. A title change to remove diversity without any revision of the data would be less misleading. | We welcome the reviewer's thoughts on other topics that could be explored in future studies. We will certainly bear these in mind. The inclusion measures chosen for this study are based on the digital divide literature, as cited in the manuscript. |  |
| 3 | Title: Diversity | Diversity and inclusion metrics could be disaggregated for gender or age as a key measure of social inclusion. Ethnicity and linguistics may or may not be relevant or easy to compare given the two nations lack of direct comparison; however, the survey was entirely completed in English. Differences between events, and those speaking English as a first or second language at both events may reveal other influences on audience participation, socio-economic status and or other barriers to accessing the event eg. disability, isolation. |  |  |
| 4 | Title: Inclusion Indicators | Quantify the number of inclusion metrics as measures are not exhaustive. 2 inclusion indicators would frame the body of work better. | Unclear what concrete changes in the manuscript are being requested by the reviewer. |  |
| 5 | Abstract: Digital Divide | Income and education are often cited with age, linguistics, race and other factors in context of digital divide. Would help to acknowledge the communication infrastructure necessary to support digital delivery, comparison between the two locations Malta/Ireland and why other indicators have not been included in the statistical measure. | This is a real-world study conducted in the context of live events. As such, the length of the survey instruments needed to be kept as brief and focused as possible. For this reason, social inclusion variables that are most strongly highlighted in the digital divide literature (cited in the manuscript) were prioritized over other variables. |  |
| 6 | Introduction: Literature | Digital divide literature review methodology would be helpful to include. | The manuscript does not claim to offer a systematic review of the literature - this would require a separate paper. |  |
| 7 | ERN Night: Marketing campaigns | Aside from spend, were the demographic targeting settings for digital marketing the same for both? Could this have impacted the diversity of the audience / outcomes of the study? | The overall approach to digital advertising was broadly similar in both years: this has now been clarified in the manuscript. We feel a detailed account of the marketing approach for each of the European Researchers Night events analysed would be beyond scope of the current manuscript. |  |
| 8 | Discussion: COVID-19 | Given the pre and post-pandemic setting for the study, what relevance does it bring to bear on the digital divide and it’s impacts on diverse audiences’ engagement with science? | The pandemic context is now explicitly flagged in the Discussion section, as follows: '' In this paper, the profiles of audiences engaging with online and offline public engagement with research events were compared, *taking advantage of the natural experiment conditions created by the pandemic*.” |  |

| Reviewer #3 | | | |
| --- | --- | --- | --- |
| 1 | Overall | This study provides an interesting ‘natural experiment’ comparing in-person and online attendance at research engagement events pre- and mid-COVID pandemic (2019 vs 2020). This is a well-presented manuscript which will be of interest to many event organisers who continue to be impacted by COVID-19 in their ability to fully present in-person events and may have valid concerns about their ability to reach certain audiences online. The authors have done well to connect their results to the so-called ‘digital divide’, and with a few minor changes will be able to publish a study that has wide significance across numerous fields. | No changes requested. |
| 2 | Minor comments: Key words | The keywords provided are limited and repetitive: I recommend adding “science communication” or science/research engagement/outreach or “public engagement in science (or research)” or likewise to help researchers in those fields find this study when it is published. | Have added ‘science communication’ to the list of keywords. No other changes are requested. |
| 3 | Tables | Tables 1, 2 and 3 use the terms ‘enrolled’ and ‘achieved’ but these are not easy to understand out of context and do not appear well defined in text or in the table captions. Perhaps it would be cleared to use ‘recruited’ or ‘invited’ and ‘responded’, which would align more closely with the description in the methods. | We are grateful for this suggestion and have updated the manuscript accordingly to be ''invited'' and ''responded''. |
| 4 | Tables | From the in-text description it is clear which city ‘Case 1’ and ‘Case 2’ refers to, so I am unclear why they cannot simply be referred to by the city in the table, i.e. Dublin 1 and Dublin 2. It seems to introduce an unnecessary element of confusion to use different labels in the table when they are clearly identified elsewhere. | We felt it would be simpler to present the results at a national level in general but that the tables needed to differentiate by institution/location to be technically accurate in case future researchers may want to use these data for meta-analyses. This is the rationale for the current presentation. In particular, our judgment is that Dublin 1 / Dublin 2 / Cork would be a more confusing labelling for readers than the current phrasing of Case 1, Case 2, Case 3. We appreciate this is a judgment call though so if PLOSONE Editors have a strong view, we are happy to update the table accordingly. |
| 5 | Chi-square tests | For the chi-square test of hypothesis 2, no significant effect has been detected and I think the current wording ‘an extremely weak statistical relationship’ is misleading. Please rephrase to make it clear no relationship was detected between economic wellbeing and year. However, you might consider running the test on Ireland alone to see if there is a difference when you separate country. Is there any economic difference between Ireland and Malta that would contribute to a different result? | We are grateful to the reviewer for highlighting this for our attention. We have amended this section accordingly. |
| 6 | Data availability | In response to Q3 – have the authors made data full available: In the authors’ responses they have indicated data from the study will be available on request, whereas the PLOS Data Policy requires data to be fully available as part of the manuscript, supporting information or in a public repository. The authors should make their data available in such a manner, or explain why they are unable to meet the policy’s requirements. | In line with PLOS ONE policy, we will make the open data available on Zenodo prior to publication. |
| 7 | Ethics statement | Is there an approval number from the Trinity College ethics committee? This statement is quite vague: “an ethics committee” – is there more than one? | There is no reference number in the Irish system. They just send a brief approval email with no unique ID visible. |
| 8 | Major comments: | The introduction to the ERN events is generally very good and provides sufficient background to non-European readers. However, it might be useful to know if the European Commission has particular aspirations in terms of who is reached through these events, for instance those with less formal education or scientific qualifications. If such goals exist, it would be useful context in which to read this study’s findings and could be linked in the discussion in terms of whether a shift to online helped or hindered the reaching of these particular audiences. | This is already addressed in the first section of **European Researchers’ Night: Background and Changing Context**. |
| 8 | Event cost | It is not clear from the manuscript whether ERN events are free or ticketed. This is relevant information since moving events online would have a likely impact on cost (if there is any cost to attendees). Please amend the manuscript to make this distinction clear: if events are free, it can be mentioned early and dismissed as a potential factor influencing attendance. If there is a cost to attendees, then this should be considered as part of the implications of moving online and should be canvassed in the discussion. | This is now addressed in the first section of **European Researchers’ Night: Background and Changing Context** with an additional sentence: “*These events were free of charge to all attendees in both 2019 and 2020*.” |
| 9 | Discussion: Digital divide | The discussion section is limited and based mostly around the ‘digital divide’, however, there are several interesting avenues the authors could discuss their findings in light of. For instance, for the Ireland events in particular it appears there was a focus on in-person events held on university campuses: there is currently no discussion on whether the shift to online may have facilitated a greater breadth of attendees by democratising the venue and being more open to attendees with less formal education who otherwise might feel uncomfortable at a university-based event. | We have added the following sentence to the Discussion to drive home this important point: “*These findings suggest the possibility that moving public engagement with research online may have had a democratizing effect on participation.*” The reason we do not focus too intensively on the university campus point is that in Malta (where the effect of the move online was greatest) there were a mix of locations used, mostly not on campuses but rather in the city centre. In Ireland, where most activities were on university campuses, the changes associated with the move online was much smaller. |
| 10 | Discussion: Recommendations | Currently missing from the discussion is any form of recommendation or reassurance for those running such outreach events. It would be useful to comment on such events in the context of these results, especially as some event organisers may choose to operate ‘dual mode’ or provide some online events to help attract a wider audience. It would also be interesting to discuss whether delivering events online was cost-effective to organisers and funders as a way of reaching a broader range of attendees perhaps in addition to in-person events. | We appreciate the excellent suggestions from the reviewer here. However, we feel these practical topics are beyond the scope of our scientific paper, and risk drawing us into the realm of opinion here. We are of course keen to engage on these practical dimensions, and are doing so with an upcoming webinar for event organizers and a planned practice-oriented commentary as a follow-up (where the co-authors on this paper who are involved in organizing events will be leading). |
| 11 | Discussion: Findings | Overall, I think the authors haven’t done their study justice in the discussion: their findings raise several interesting points about whether online delivery might attract a wider range of attendees to such events and better achieve goals to connect with audiences with less formal education. At least this should be discussed, potentially in the form of recommendations for event organisers or further research examining events that were forced online in 2020/21. This could also be better tied back to science/research outreach and the goal of connecting with a broader range of audiences. |  |

| Reviewer #4 | | | |
| --- | --- | --- | --- |
| 1 | Overall | The manuscript is technically sound, well-written, and all data discussed are clearly presented. | No changes requested. |
| 2 | Statistics | The statistics need some further explanation to ensure that they have been rigorously tested. As an example, you refer to four 'cases' but it is not clear how these were tested statistically, and how classifying these into 4 cases is important. | We felt it would be simpler to present the results at a national level in general but that the tables needed to differentiate by institution/location to be technically accurate in case future researchers may want to use these data for meta-analyses. This is the rationale for the current presentation. In particular, our judgment is that Dublin 1 / Dublin 2 / Cork would be a more confusing labelling for readers than the current phrasing of Case 1, Case 2, Case 3. We appreciate this is a judgment call though so if PLOSONE Editors have a strong view, we are happy to update the table accordingly. |
| 3 |  | Overall, the paper demonstrates an important finding, that shifting public science events online can polarise the audience by strengthening attendance from higher educated groups whilst also attracting those with no formal qualification. | No changes requested. |
| 4 | Discussion | I felt that the manuscript was rather too simplistic in this analysis however, as there was no discussion about other interesting demographics and how these may be affected by a move to an online delivery. | This is a real-world study conducted in the context of live events. As such, the length of the survey instruments needed to be kept as brief and focused as possible. For this reason, social inclusion variables that are most strongly highlighted in the digital divide literature (cited in the manuscript) were prioritized over other variables. |
| 5 | Case selection | It was also not explained why Malta and Ireland were chosen for this study. | Malta and Ireland were selected as the focus for this study because coordinators of these ERN events agreed to collaborate on this research. |
| 6 | Recommendations | No recommendations were given for improving online events to attract a wider demographic. | We appreciate the excellent suggestions from the reviewer here. However, we feel these practical topics are beyond the scope of our scientific paper, and risk drawing us into the realm of opinion here. We are of course keen to engage on these practical dimensions, and are doing so with an upcoming webinar for event organizers and a planned practice-oriented commentary as a follow-up (where the co-authors on this paper who are involved in organizing events will be leading). |
| 7 | Recommendations | I would be interested to see a revised version that presents evidence for future event organisers (and other science communication practitioners) to use to ensure greater representation of diverse society at these events. |  |

Further Revisions:

1. Thank you for including your ethics statement on the online submission form: "This research received ethical approval from an ethics committee at Trinity College Dublin.". To help ensure that the wording of your manuscript is suitable for publication, would you please also add this statement at the beginning of the Methods section of your manuscript file.

[This line has now been added to the manuscript]

2. Please amend the title either on the online submission form or in your manuscript so that they are identical.

[The online submission title has been changed to match the manuscript title]

3. Please include a copy of Table 4.

[Table 4 is now included]

4. We note your current Data Availability statement: "The data underlying the results presented in the study are available from the authors on request."

[The Data Availability statement has now been changed to say: "The anonymized dataset underlying the results presented in the study will be published on the open-access database Zenodo following acceptance and prior to publication, in line with PLOS ONE data accessibility policy."]
